# Flame-Spray-Made Undoped Zinc Oxide Films for Gas Sensing Applications

**DOI:** 10.3390/s100807863

**Published:** 2010-08-23

**Authors:** Nittaya Tamaekong, Chaikarn Liewhiran, Anurat Wisitsoraat, Sukon Phanichphant

**Affiliations:** 1 Nanoscience Research Laboratory, Department of Chemistry, Faculty of Science, Chiang Mai University, Chiang Mai, 50200, Thailand; E-Mail: doramon_koygy@hotmail.com (N.T.); 2 Department of Materials Science, Faculty of Science, Chiang Mai University, Chiang Mai, 50200, Thailand; E-Mail: chaikarn_l@yahoo.com (C.L.); 3 National Electronics and Computer Technology Center, Pathumthani, 12120, Thailand; E-Mail: anurat.wisitsoraat@nectec.or.th (A.W.)

**Keywords:** undoped ZnO, flame spray pyrolysis, NO_2_, C_2_H_5_OH, SO_2_, gas sensor

## Abstract

Using zinc naphthenate dissolved in xylene as a precursor undoped ZnO nanopowders were synthesized by the flame spray pyrolysis technique. The average diameter and length of ZnO spherical and hexagonal particles were in the range of 5 to 20 nm, while ZnO nanorods were found to be 5–20 nm wide and 20–40 nm long, under 5/5 (precursor/oxygen) flame conditions. The gas sensitivity of the undoped ZnO nanopowders towards 50 ppm of NO_2_, C_2_H_5_OH and SO_2_ were found to be 33, 7 and 3, respectively. The sensors showed a great selectivity towards NO_2_ at high working temperature (at 300 °C), while small resistance variations were observed for C_2_H_5_OH and SO_2_, respectively.

## 1. Introduction

Zinc oxide has attracted increased attention during the last few years due to the possibility of its relatively simple transformation into various nanoscale structures. Nanostructures like rods and particles have become the most promising research materials because of their wide range of applications. Different techniques, namely sol-gel [1], spray pyrolysis [2], hydrothermal method [3,4], electrospinning [5], thermal evaporation [6,7], *etc.* [8–13] are prevalent for the synthesis of zinc oxide nanoparticles and nanorods. In the present work, nanorods and nanoparticles have been prepared by flame spray pyrolysis (FSP), a promising technique for the synthesis of high purity nano-sized materials with controlled size and crystallinity in a single step. This was systematically investigated by using an external-mixing gas-assisted atomizer supported by six premixed methane-oxygen flamelets [14].

Semiconducting metal oxide sensors have been extensively studied due to their simple preparation and high sensitivity under ambient conditions [15–20]. Zinc oxide (ZnO), an n-type metal oxide semiconductor sensing material with a wide band gap (*E*g = 3.37 eV at 300 K), has attracted much attention due to its high chemical stability, low cost, and good flexibility in fabrication. It was found that ZnO exhibits pronounced gas sensing properties towards many toxic/non-toxic gases such as NO_2_, SO_2_, ethanol, *etc*. [21–31]. A summary on the sensing properties toward NO_2_, ethanol (C_2_H_5_OH) and SO_2_ gases of the undoped ZnO prepared by several synthetic methods is shown in Table 1.

Great interest in improving the gas sensitivity as well as selectivity and in decreasing the working temperature has been witnessed. Nitrogen dioxide (NO_2_) is considered a common air pollutant produced during combustion in automotive engines, industrial factories, and power plants. Therefore, the development of stable NO_2_ gas sensors that can detect extremely low concentrations of NO_2_ with high sensitivity is highly desirable [32]. In this study, undoped ZnO nanopowders have been prepared by the flame spray pyrolysis process and their gas sensing responses towards different gases have been comparatively examined. In particular, three types of sensors were tested under oxidizing and reducing gases, like nitrogen dioxide, ethanol and sulfur dioxide.

## 2. Experimental

### 2.1. Particle synthesis and characterization

Zinc naphthenate (Aldrich, 8 wt% Zn) was used as a precursor. The precursor was dissolved in xylene (Carlo Erba, 98.5%) to obtain a 0.5 mol/L precursor solution. In a typical run, the precursor was fed into a FSP reactor by a syringe pump with a rate of 5 mL/min while 5 L/min O_2_ was being dispersed (5/5 flame). The gas flow rates of methane and O_2_ supporting flamelets were 1.19 and 2.46 L/min, respectively. The pressure drop at the capillary tip was kept constant at 1.5 bars by adjusting the orifice gap area at the nozzle.

The flame height was observed to be approximately 10–12 cm. The sample showed a yellowish-orange flame. The liquid precursor mixture was rapidly dispersed by a gas stream and ignited by a premixed methane/oxygen flame. After evaporation and combustion of precursor droplets, particles are formed by nucleation, condensation, coagulation and coalescence. Finally, the nanoparticles were collected on glass microfiber filters with the aid of a vacuum pump. The undoped ZnO nanopowders were characterized by X-ray diffraction (XRD), scanning electron microscopy (SEM) and transmission electron microscopy (TEM). Specific surface area (*SSA*_BET_) of the nanoparticles was also investigated by nitrogen adsorption (BET analysis).

### 2.2. Sensing film preparation and characterization of the gas sensing properties

The undoped ZnO sensing film was prepared by mixing the nanoparticles into an organic paste composed of ethyl cellulose and terpineol, which acted as a vehicle binder and solvent, respectively. The resulting paste was spin-coated on Al_2_O_3_ substrates with predeposited interdigitated Au electrodes. The films were then annealed at 400 °C for 2 h (with heating rate of 2 °C/min) for binder removal. The morphology and the cross section of sensing films were analyzed by SEM.

The gas-sensing characteristics of the undoped ZnO nanoparticles towards NO_2_, C_2_H_5_OH and SO_2_ were characterized. The flow through technique was used to test the gas-sensing properties of thin films. A constant flux of synthetic air of 2 L/min was mixed with desired concentrations of pollutants. All measurements were conducted in a temperature-stabilized sealed chamber at 20 °C under controlled humidity. The external NiCr heater was heated by a regulated dc power supply to different operating temperatures. The operating temperature was varied from 200 °C to 350 °C. The resistances of various sensors were continuously monitored with a computer-controlled system by voltage-amperometric technique with 5 V dc bias and current measurement through a picoammeter. The sensor was exposed to a gas sample for ∼5 minutes for each gas concentration testing and then the air flux was restored for 15 minutes. The concentration of NO_2_, C_2_H_5_OH and SO_2_ were varied from 1 to 50 ppm, 50 to 100 ppm and 10 to 500 ppm, respectively.

## 3. Results and Discussion

### 3.1. Particle properties

**Figure 1.**
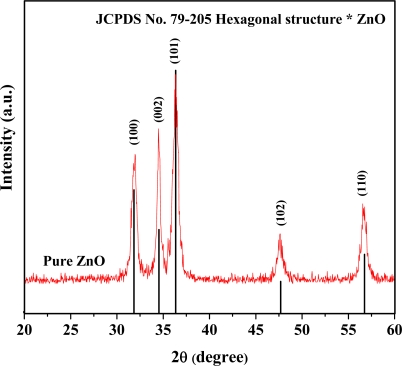
The XRD patterns of flame-spray-made (5/5) undoped ZnO nanopowders.

Figure 1 shows the XRD patterns of the undoped ZnO sample. All peaks can be confirmed to correspond to the hexagonal structure of ZnO (JCPDS No. 79-205).

An average BET equivalent particle diameter (*d*_BET_) was calculated using the average density of ZnO as shown in Table 2. The accurate particle size and morphology of undoped ZnO dispersion were confirmed by SEM and TEM images.

**Figure 2.**
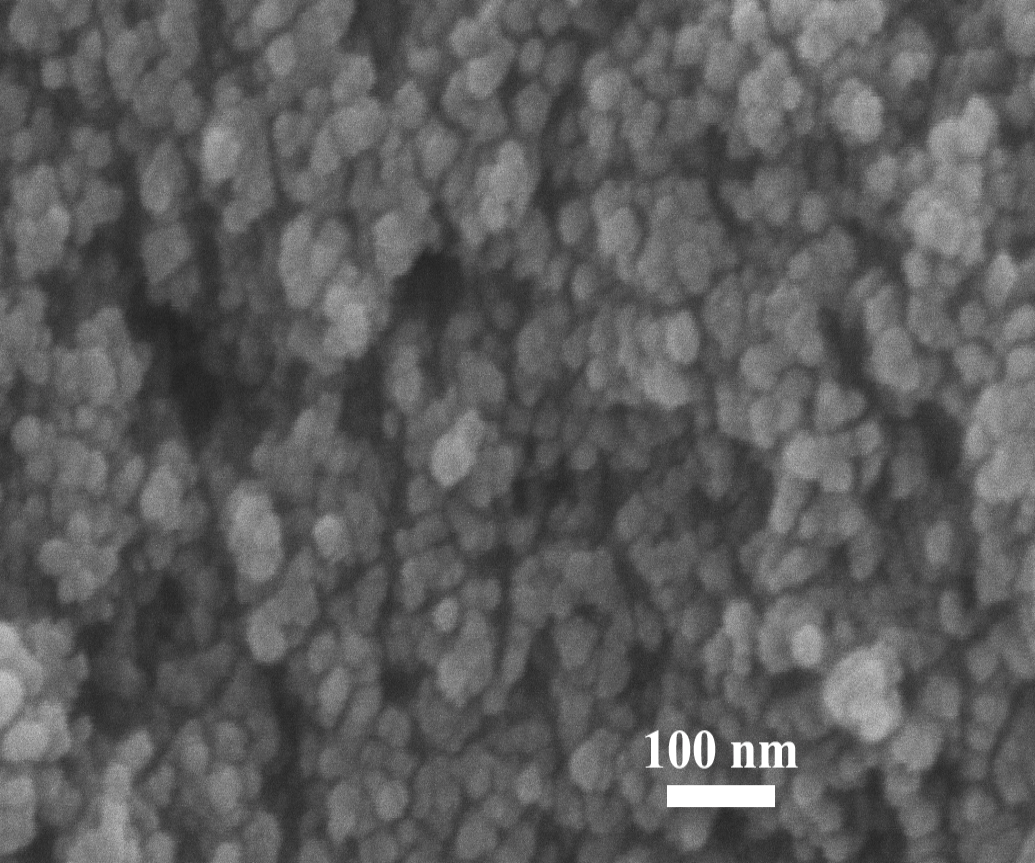
The SEM micrograph of highly crystalline flame-made (5/5) undoped ZnO nanoparticles.

Figure 2 shows the morphology of highly crystalline flame-made (5/5) undoped ZnO nanoparticles from SEM analysis. The SEM micrograph clearly showed nanostructural homogeneities and remarkably different morphologies of the undoped ZnO nanoparticles synthesized by the FSP technique. The SEM result showed the presence of agglomerated nanospheres with an average diameter of 10–20 nm. Therefore, from this observation only the rough morphology was found. Nevertheless, the accurate sizes and morphology of the nanoparticles can be estimated from the TEM analysis. While the SEM images provide 3-D morphology and estimated particle sizes, TEM images can reveal internal structure and a more accurate measurement of particle size and morphology.

**Figure 3.**
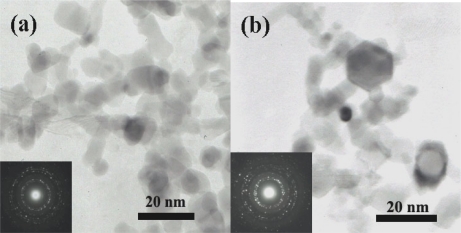
The TEM images of undoped ZnO morphologies showing the spherical, hexagonal and rod-like shapes.

Figure 3 shows the TEM-bright-field images of undoped ZnO nanoparticles. The ZnO morphologies were revealed to be spherical, hexagonal and rod-like. The presence of ZnO spherical nanoparticles along with a few nanorods was observed as shown in Figure 3(a). The crystallite sizes of spherical particles were found to be in the range of 5–20 nm whereas the nanorods were found to be ranging from 5–20 nm in width and 20–40 nm in length. Hexagonal ZnO nanoparticles with the size of 5–20 nm were also observed, as shown in Figure 3 (b).

### 3.2. SEM sensing layer

**Figure 4.**
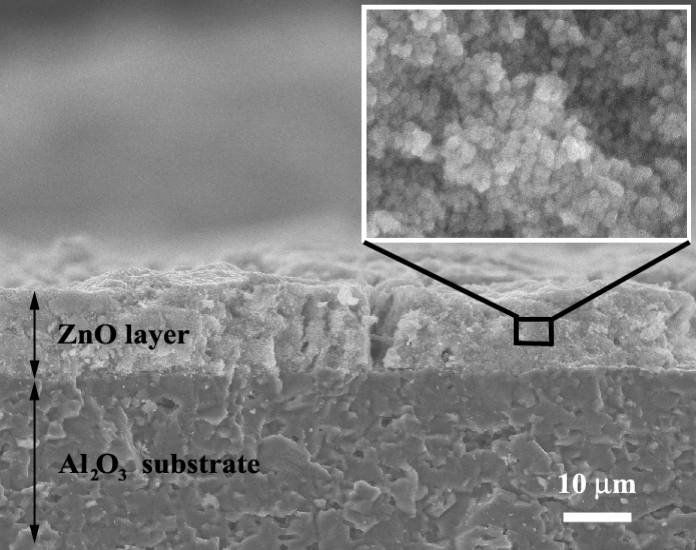
The SEM micrographs of flame-made undoped ZnO thick films sensor on an Al_2_O_3_ substrate interdigitated with Au electrodes after annealing and sensing test at 300 °C in dry air. The film thickness was approximately 10 μm.

The cross-section, film thickness, and surface morphology of the undoped ZnO sensing film layer after annealing and sensing test at 300 °C were observed using SEM analysis, as shown in Figure 4. The thickness of sensing film was approximately 10 μm (side view) which benefited tremendously the NO_2_, C_2_H_5_OH and SO_2_ gas sensing properties. Irregularities in the film thickness (top view) stem from the spin coating technique. The high density Al_2_O_3_ substrate interdigitated with Au electrodes was also visible. After the annealing process, a denser film layer was formed.

**Figure 5.**
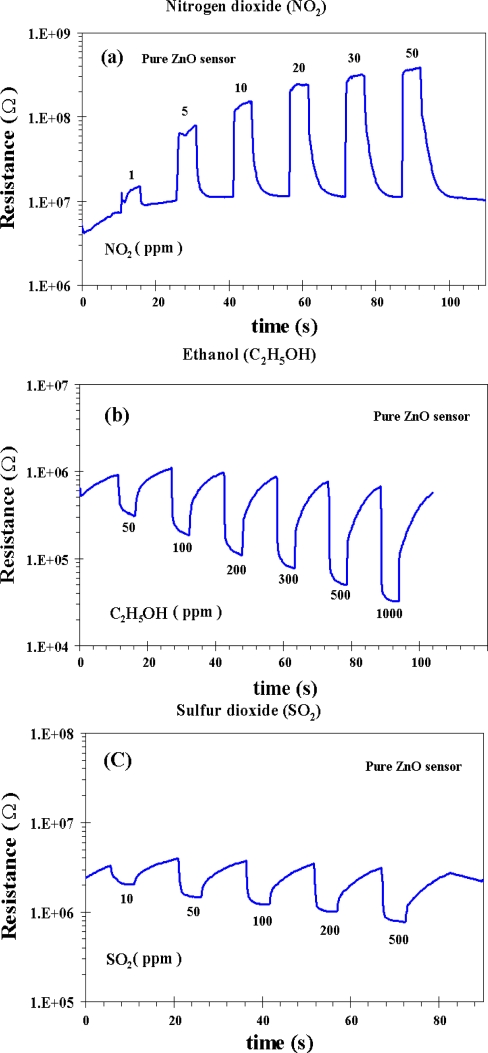
The change in resistance of undoped ZnO sensor for NO_2_ **(a)** C_2_H_5_OH **(b)** and SO_2_ **(c)** gases under exposure to oxidizing gas of NO_2_ and reducing gases of C_2_H_5_OH and SO_2_ during forward cycle at 300 °C, respectively.

The sensitivity and response time of the thick films of the undoped ZnO nanoparticles as a function of NO_2_, C_2_H_5_OH and SO_2_ concentrations at 300 °C are shown in Figure 5. In Figure 5(a), it can be seen that the sensitivity toward NO_2_ is increased considerably at 50 ppm NO_2_ concentration. The sensitivity and response time for the undoped ZnO nanoparticles at 50 ppm NO_2_ concentration were found to be 33 and 7 s, respectively. The sensitivity, however, are decreased considerably by testing the undoped ZnO sensor with C_2_H_5_OH and SO_2_ at 50 ppm concentration of each gas. The sensitivity of 7 and 3 with the response time of 94 and 17 s are obtained at 50 ppm of C_2_H_5_OH and 50 ppm of SO_2_, respectively. It is important to note that the undoped ZnO nanoparticles behave as an n-type semiconductor with decreased resistance during NO_2_, C_2_H_5_OH and SO_2_ gas exposure, which is a typical behavior of ZnO material [33]. The gas-sensing sensitivity, *S*, is defined as the ratio of *R_a_/R_g_* where *R_a_* is the resistance in dry air, and *R_g_* is the resistance in test gas. The response time, *T*_res_, is defined as the time required until 90% of the response signal is reached. The recovery time, *T*_rec_, denotes the time needed until 90% of the original baseline signal is recovered. The sensor behaviors under the operating temperature of 300 °C versus the NO_2_ concentrations ranging from 1–50 ppm for the flame-made undoped ZnO nanoparticles were plotted as shown in Figure 5(a). The changes in resistance of the undoped ZnO sensor for C_2_H_5_OH and SO_2_ gases under exposure to 50–1,000 ppm of C_2_H_5_OH and 10–500 ppm of SO_2_ during forward cycle at 300 °C are shown in Figures 5(b,c), respectively.

**Figure 6.**
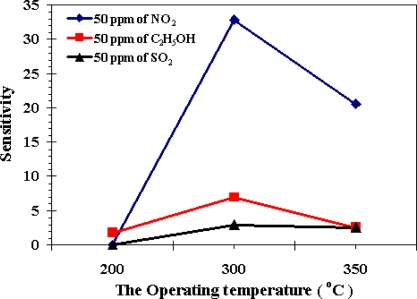
The sensitivity *versus* variation of the operating temperature of NO_2_, C_2_H_5_OH and SO_2_ (at 50 ppm of concentration) for the undoped ZnO sensor.

It is well known that the sensitivity of a semiconductor gas sensor is highly influenced by its operating temperature [34,35]. In order to determine the optimum operating temperatures, the response of the undoped ZnO gas sensor to 50 ppm concentration of nitrogen dioxide, ethanol and sulfur dioxide in air was tested as a function of operating temperature, as shown in Figure 6. It is clear that the responses of three gases tested varied with operating temperature. The sensitivity to NO_2_ first increased with temperature, up to 300 °C, and then gradually decreased. The maximum sensitivity towards NO_2_ was 33, at 300 °C. For ethanol and SO_2_, the sensitivity continuously increased when operating temperatures varied from 200 to 300 °C, and then decreased. The maximum sensitivities obtained were 7 and 3, at 300 °C. Therefore, optimal operating temperatures of 300 °C were chosen for NO_2_, ethanol and SO_2_ respectively, to further examine the characteristics of the gas sensor. Results suggest that the undoped ZnO sensor can act as a multifunctional selective gas sensor, detecting NO_2_, ethanol and SO_2_ gases. In other words, the above mentioned sensor can be used as an excellent NO_2_ sensor at an operating temperature of 300 °C.

**Figure 7.**
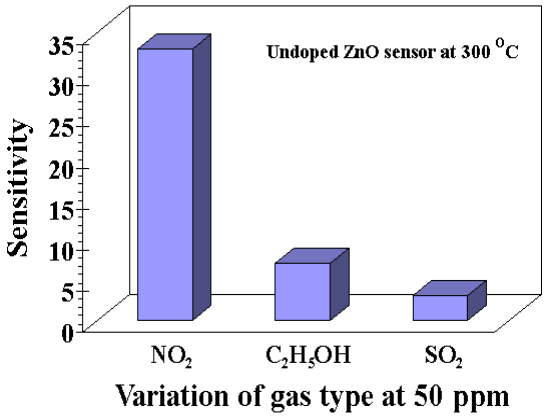
The sensitivity variation of the undoped ZnO sensor testing with 50 ppm concentration of NO_2_, C_2_H_5_OH and SO_2_ under the operating temperature of 300 °C.

The gas sensing selectivity of the undoped ZnO gas sensor has been characterized towards one common oxidizing gas, nitrogen dioxide (NO_2_), and two other common reducing gases, ethanol (C_2_H_5_OH) and sulfur dioxide (SO_2_) as shown in Figure 7. In Figure 7, the sensitivities towards NO_2_, C_2_H_5_OH and SO_2_ under the operating temperature of 300 °C were found to be 33, 7 and 3, respectively. This indicates an excellent NO_2_ selectivity of our undoped ZnO gas sensor.

The sensitivities of the flame-made ZnO sensor towards different concentrations of NO_2_, C_2_H_5_OH and SO_2_ gases obtained from our studies are summarized in Table 3. In comparison with the previously reported sensitivities of other ZnO sensors given in Table 1 [21–24], the flame-made ZnO films showed higher sensitivity towards the same NO_2_ concentration in all cases. Sensitivity of flame-made ZnO films towards 100 ppm of SO_2_ was 2.8, whereas the result from Lupan *et al*. [26] was less than 0.5. Likewise flame-made ZnO films showed sensitivity towards 200, 300 and 500 ppm of ethanol as 18.2, 22.4 and 27.5 respectively which were higher than the values reported by Singh *et al.* [28] and Hieu *et al.* [31].

## 4. Conclusions

In summary, we have shown that FSP is a promising technique for the synthesis of high purity nano-sized materials with controlled size and crystallinity in a single step, exemplified by the fabrication of an undoped ZnO array sensor that can sense NO_2_, C_2_H_5_OH and SO_2_ gases. The undoped ZnO-based NO_2_ gas sensor showed the lowest detection limit of 1 ppm with short response and recovery time. Moreover, the sensors showed a high selectivity towards NO_2_ at 300 °C when compared with C_2_H_5_OH and SO_2_ gases, respectively. The sensitivity of undoped ZnO film towards 50 ppm of NO_2_, C_2_H_5_OH and SO_2_ were 33, 7 and 3 respectively.

## Figures and Tables

**Table 1.** A summary on the gas sensing properties of differently-prepared undoped ZnO for NO_2_, ethanol (C_2_H_5_OH) and SO_2_ gases.

**Authors [ref.]**	**Method**	**Nanoparticles**	**Gas concentration**	**Sensitivity**
**Ghimbeu *et al*. [21]**	Electrostatic spray deposition (ESD) technique	Undoped ZnO	1 ppm of NO_2_, at 300 °C	∼1.84
**Cho *et al.* [22]**	Hydrothermal	Undoped ZnO	1 ppm of NO_2_, at 300 °C	∼1.8
**Sadek *et al.* [23]**	Conventional solid-state method	Undoped ZnO	10 ppm of NO_2_, at 350 °C	∼1.81
**Lupan *et al.* [26]**	A solution method	Undoped ZnO	100 ppm of SO_2_	<0.5
**Singh *et al.* [28]**	A simple chemical route	Undoped ZnO	250 ppm of ethanol, at 400, 600 and 800 °C	∼6.5, 5.6 and 4,
**Hieu *et al.* [31]**	Thermal evaporation	Undoped ZnO	500 ppm of ethanol, at 300 °C	5.3
**Present work**	Flame spray pyrolysis (FSP)	Undoped ZnO	1, 5 and 10 ppm of NO_2_, at 300 °C	∼2.7, 6.2 and 11.8
			100 ppm of SO_2_, at 300 °C	∼2.8
			200, 300 and 500 ppm of ethanol, at 300 °C	∼18.2, 22.4 and 27.5

**Table 2.** The specific surface area (*SSA*_BET_) and *d*_BET_ of undoped ZnO nanoparticles.

**Sample**	**Specific surface area (*SSA*_BET_), (m^2^/g)**	***d*_BET_ (nm)**
Undoped ZnO	78.8	13.6

**Table 3.** Sensitivity of flame-made undoped ZnO nanoparticles towards different concentrations of NO_2_, C_2_H_5_OH and SO_2_ gases under the operating temperature of 300 °C.

**Gas concentration**	**Sensitivity**
1, 5, 10, 20, 30 and 50 ppm of NO_2_	2.7, 6.2, 11.8, 18.5, 26.7 and 33
100 ppm of SO_2_	2.8
200,300, and 500 ppm of ethanol	18.2, 22.4 and 27.5
